# Efficacy of Combined Conjoint Fascial Sheath and Levator Muscle Composite Flap Suspension for Recurrent Severe Ptosis

**DOI:** 10.1007/s00266-025-04838-4

**Published:** 2025-04-16

**Authors:** Huixing Wang, Zhaochuan Liu, Junhu Shi, Hongbin Zhang, Shan Liu, Yan Tan, Congying Feng, Runhui Pang, Ping Bai

**Affiliations:** 1 Department of Ocular Plastic, Hebei Eye Hospital, Hebei Provincial Key Laboratory of Ophthalmology, Hebei Provincial Clinical Research Center for Eye Disease, Xingtai, 054000 Hebei China; 2 Department of Ophthalmology, Beijing Tongren Hospital, Beijing Ophthalmology and Visual Science Key Lab, Capital Medical University, Beijing, 100730 China

**Keywords:** Ptosis, Recurrence, Conjoint fascial sheath

## Abstract

**Background:**

Conjoint fascial sheath (CFS) and levator muscle (LM) composite flap suspension has been gradually accepted for the treatment of congenital severe blepharoptosis in recent years. This study is to evaluate the clinical efficacy of conjoint fascial sheath with levator muscle (CFS + LM) suspension in the treatment of recurrent severe ptosis.

**Methods:**

A total of 45 patients (51 eyes) with recurrent severe ptosis admitted to our hospital between February 2018 and October 2022 were treated with CFS + LM suspension. The surgical efficacy was evaluated by recording and analyzing the distance (in millimeters) from the pupillary reflex on the patient’s cornea to the upper eyelid margin (MRD1), palpebral fissure height (PFH), lagophthalmos (LAG), degree of satisfaction and complications.

**Results:**

A total of 45 patients (51 eyes) with recurrent severe ptosis were included in this study, including 26 males and 19 females, with an average age of 23.16 ± 10.68 years. The average levator function was 1.98 ± 1.35 mm, and the average postoperative follow-up time was 19.71 ± 13.77 month. The preoperative MRD1 and PFH were − 0.18 ± 1.03 mm and 3.90 ± 0.88 mm, respectively. Postoperative follow-up was conducted at least 6 months, and the MRD1 and PFH values were 2.25 ± 0.77 mm and 7.04 ± 0.82 mm at the last follow-up, respectively; the differences with the corresponding preoperative values were significant (*p*< 0.05). All the operated eyes had different degrees of LAG, which also stabilized 6 months after surgery. The treatment was considered curative for 30 eyes (58.82%), to have made improvement in 11 eyes (21.57%), and ineffective for 10 eyes (19.61%). The overall effectiveness (curative + improvement) rate was 80.39%, and the total degree of satisfaction reached 77.78%. After surgery, conjunctival prolapse occurred in 3 patients, 2 of whom recovered after placement of fornix sutures. Conjunctival prolapse in 1 patient disappeared after conservative treatment. Exposure keratitis occurred in 1 patient and improved after drug treatment. No other complications occurred.

**Conclusions:**

CFS + LM suspension technique for correcting recurrent severe ptosis is easy to perform, causes minimal trauma, and results in good therapeutic effects and few complications. We recommend popularizing this technique in clinical practice.

**Level of Evidence II:**

This journal requires that authors assign a level of evidence to each article. For a full description of these Evidence-Based Medicine ratings, please refer to the Table of Contents or the online Instructions to Authors www.springer.com/00266.

**Supplementary Information:**

The online version contains supplementary material available at 10.1007/s00266-025-04838-4.

## Introduction

Upper eyelid ptosis is the incorrectly low position of the upper eyelid, which results in a limited visual feld and the deterioration of visual acuity [1–3]. Ptosis can be divided into three grades according to its severity: mild, moderate and severe. Severe ptosis is often the most difficult problem due to postoperative recurrence and poor eyelid closure. In the past, frontalis muscle flap suspension was typically used for severe ptosis [4], but this has consistently been followed by poor eyelid excursion, severe lagophthalmos and unnatural eyelid contour [5–8]. For patients with recurrence after surgery, if frontalis muscle flap suspension is performed again, it not only increases the difficulty of surgery, but also increases the scope of surgical trauma, making it difficult to achieve good results. Combined fascia sheath (CFS) was from the classic article of anatomy published in 1932 by Dr Whitnall. It was a special structure referring to“conjoint fascial sheath”of the levator and superior rectus attached to the conjunctival fornix. A modified approach of CFS and LM complex suspension (modified CFS + LM suspension) to correct severe ptosis has attracted much attention in recent years, and achieved good clinical results [9, 10]. It has been gradually recognized in clinical practice because of its minimal trauma and good postoperative upper eyelid excursion. However, there are few reports on the repair of recurrent poor function ptosis. Since 2018, our department has used the CFS + LM suspension technique to repair recurrent severe ptosis and has achieved good clinical results, as reported below.

## Materials and Methods

### Patients

Data from 45 patients (51 eyes) with recurrent severe ptosis, including 26 males and 19 females, were collected. All patients were treated via CFS + LM suspension. A total of 39 patients underwent unilateral correction, and 6 underwent bilateral correction. The mean age was 23.16 ± 10.68 years (range, 13–56 years). Levator function (LF) was measured by Berke’s method, which calculated the height difference of the upper eyelid margin with the patient looking lowest and highest after the surgeon has pressed the eyebrows to stop the function of the frontalis muscle [11, 12]. The criteria of congenital severe ptosis were that LM function was no more than 4 mm or less, the upper palpebral margin covered 1/2 of the pupil, and the amount of ptosis was > 4 mm. Elevation test was performed in all patients with ptosis before correction to determine the presence of Hering's phenomenon. The inclusion criteria were as follows: (1) congenital severe ptosis and one or more previous ptosis correction surgeries; (2) follow-up of at least 6 months; and (3) good eyeball rotation in all directions. The exclusion criteria included myasthenia gravis, Marcus Gunn jaw–winking syndrome (MGJWS), traumatic ptosis and other systemic diseases that affect the movement of the upper eyelid, dry eye syndrome and corneal lesions. Our study was approved by the Hospital Ethics Committee in compliance with the principles of the Declaration of Helsinki. Consent was obtained from the participants and their caregivers (legal guardians). The guardians of all the children provided written informed consent for the publication of surgical data and eye photographs.

### CFS + LM Suspension Technique

**Fig. 1:**
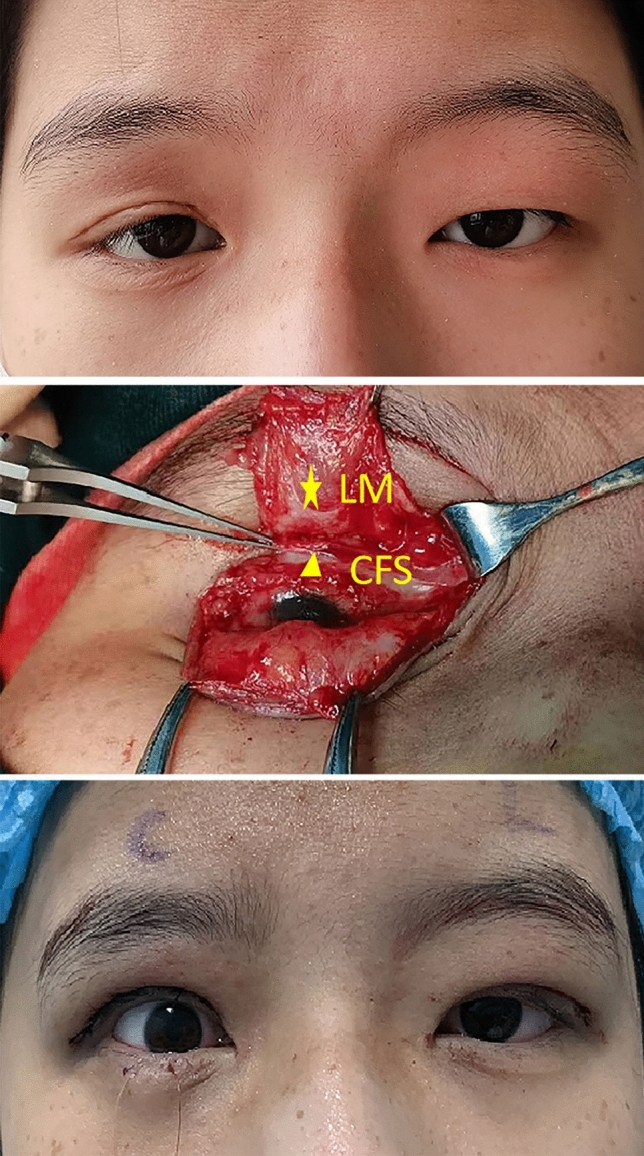
A 18-year-old girl was diagnosed with recurrent severe ptosis in right eye and congenial ptosis in left eye. The LM function was 1 mm in the right eye and 7 mm in the left eye. CFS + LM suspension was performed on the right eye, and shortening of the LM was performed in the left eye. (Above) Preoperative image. (Center) Demonstration of LM and CFS during surgery. (Below) Immediate postoperative image

All procedures were performed by the same group with the patients under local anesthesia. The operation was conducted under a microscope. (See Video 1, which demonstrates surgical procedures for CFS + LM suspension.) First, the surgical incision was designed as a double eyelid line incision and was marked in advance, and local anesthesia was administered to the upper eyelid. The skin, subcutaneous tissue and orbicularis oculi were incised sequentially along the marked line to loosen the adhesive tissue anterior to the tarsus, and the connection between the original suspender muscle flap (levator palpebral muscle flap or frontalis muscle flap) and the tarsus was dissected to expose the upper edge of the tarsus and the levator muscle (LM). Scar tissue was isolated and excised, and intraoperative release and removal of adhesive scar tissue increased bleeding, increased pain in patients, and decreased tolerance to surgery, all of which affect the separation and recognition of CFS. Therefore, intraoperative procedures should be cautious and gentle to avoid damage to normal tissue. Second, the Whitnall ligament and the medial and lateral ligaments of the levator aponeurosis were severed to relieve their limitation on the LM. Third, the eyelid was everted, and saline solution was injected for hydrodissection of the levator palpebrae superioris aponeurosis space. The levator aponeurosis was then cut open approximately 5–8 mm above the upper margin of the tarsal plate and separated upward along the levator palpebrae superioris aponeurosis space to approximately 5 mm above the fornix. The separation exposed the yellow-white fascia tissue of 3~5mm, which was located between the levator palpebral muscle and the superior rectus muscle. As shown in Fig. 1, when the patient is asked to turn the eye upward, the yellow-white fascia tissue retracts upward to confirm CFS. Then, the combined CFS + LM flap was completed. Fourth, 3 pairs of mattress sutures were placed with 5-0 absorbable sutures to fix the combined CFS + LM flap at the upper one-third site of the tarsal plate. The position of the margin of the upper eyelid is determined according to the strength of the upper eyelid levator muscle and the extension force of the upper eyelid levator muscle during the operation (amount of operation). For patients with 0–1mm LM strength, the separation height of the CFS + LM composite flap was 5–6 mm, where the upper eyelid margin was flat at the level of the upper edge of the cornea; For patients with 2–4 mm LM strength, the separation height of the composite flap was 3–4 mm, such that the upper eyelid margin covered the cornea by 0.5–1 mm. At the end of surgery, patients were positioned upright to assess the effectiveness of the procedure and the extent of lagophthalmos. Fifth, the excess levator aponeurosis was removed, and then the skin was sutured to form a double eyelid crease.

### Efficacy Criteria

We collected and recorded data including age, sex, eye laterality, follow-up time, preoperative levator muscle function (LF), preoperative MRD1 (measured in millimeters from the pupillary reflex on the patient’s cornea to the upper eyelid margin), and palpebral fissure height (PFH) of all the included patients.The surgical outcomes were categorised as curative, improved or ineffective based on the postoperative MRD1 (Table 1). Follow-up was performed 1 month, 3 months, 6 months and the last follow-up after the operation. The surgical complications included bleeding, conjunctival prolapse, palpebral separation, exposure keratitis, entropion, ectropion, and restriction of eye movement. A self-satisfaction assessment was performed by the patients themselves.

**Table 1:** Categories of surgical outcomes

Surgical outcome	Unilateral ptosis	Bilateral asymmetric ptosis
Curative	MRD1 is ≤ 1 mm compared with the opposite eye	MRD1 is ≥ 3 mm in each eye and MRD1 is ≤ 1 mm compared with the opposite eye
Improved	MRD1 is ≤ 2 mm lower than the opposite eye	MRD1 is ≥ 2 or < 3 mm and MRD1 is ≤ 2 mm lower than the opposite eye
Ineffective	MRD1 is > 1 mm (overcorrection) or < 2mm compared with the opposite eye	MRD1 is < 2 mm or > 1 mm (overcorrection) or MRD1 is > 2 mm lower than the opposite eye

MRD1, marginal reflex distance-1

### Main Outcome Measures

The patients were asked to return for follow-up at 1 month, 3 months, 6 months and the last follow-up after surgery. The postoperative MRD1, PFH and degree of lagophthalmos were measured, and the incidence of postoperative complications was recorded. Self-satisfaction was followed up and recorded.

### Statistical Methods

The above data were subjected to statistical and comparative analysis with SPSS 21.0 software. First, the normality of the distribution of the measurement data was assessed. If the data were normally distributed, comparisons between two groups were performed with the two independent sample t test. If the distribution did not conform to a normal distribution, the rank-sum test was used for between-group comparisons. Intergroup comparisons of count data were performed with the χ2 test; *P*< 0.05 was considered statistically significant.

## Results

The preoperative patient data are summarized in Table 2. In this study, a total of 45 patients (51 eyes) with recurrent severe ptosis were included, including 39 with unilateral ptosis and 6 with bilateral ptosis, with a mean age of 23.16 ± 10.68 years. Twenty-six patients were male, and 19 were female. The average follow-up time was 19.71 ± 13.77 months after surgery. The mean preoperative MRD1 was − 0.18 ± 1.03 mm, the mean PFH was 3.90 ± 0.88 mm, and the mean levator muscle function was 1.98 ± 1.35 mm. Twenty-four eyes that had previously undergone frontalis suspension, 21 eyes had levator advancement, 5 eyes underwent frontalis suture suspension, and 1 eye underwent another surgical procedure. All patients demonstrated normal eye movement before the operation, and no lagophthalmos was observed.

**Table 2:** Preoperative data of patients

Characteristics	Mean ± SD
Patients	45
Eyelids	51
Mean age (years)	23.16 ± 10.68
Sex,n	
Female/Male	19/26
Follow-up duration	
Mean ± SD months	19.71 ± 13.77
Previous correction technique	
levator advancement	21 (41.18%)
Frontalis suspension	24 (47.06%)
Frontalis suture suspension	5 (9.80%)
Other	1 (1.96%)
Laterality, n	
Unilateral /Bilateral	39/6
LF, mm	1.98 ± 1.35

LF, levator function

**Fig. 2:**
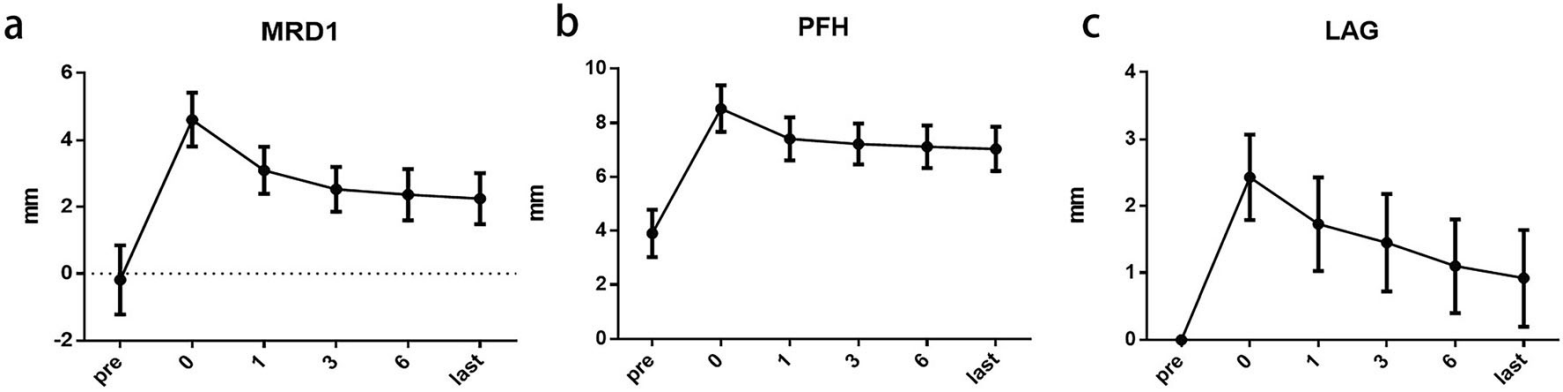
Parameter assessment of modified CFS + LM suspension. **a**, **b**, and **c**, Curve of MRD1. PFH and LAG, respectively, at 6 visit time

The MRD1 and PFH of all patients significantly improved immediately after surgery. The average MRD1 reached 4.61 ± 0.80 mm, the average PFH reached 8.53 ± 0.86 mm; moreover the eyelid curvature and suprapalpebral fold were well-formed. As summarized in Table 3, the MRD1 and PFH 1 month, 3 months, and 6 months after surgery and at the last follow-up were 3.10 ± 0.70 mm, 2.53 ± 0.67 mm, 2.37 ± 0.77 mm, and 2.25 ± 0.77 mm and 7.41 ± 0.80 mm, 7.22 ± 0.76 mm, 7.12 ± 0.79 mm, and 7.04 ± 0.82 mm, respectively. Although the MRD1 and PFH values gradually decreased after surgery, compared with the preoperative levels, both values were significantly elevated at all postoperative time points (*p*< 0.05) (Fig. 2a, b).

**Table 3:** Characteristics of MRD1, PFH, and LAG

Characteristics	Preoperative	Immediate Postoperative	1 mo	3 mo	6 mo	Last Follow-up
MRD1, mm	− 0.18 ± 1.03	4.61 ± 0.80	3.10 ± 0.70	2.53 ± 0.67	2.37 ± 0.77	2.25 ± 0.77*
PFH, mm	3.90 ± 0.88	8.53 ± 0.86	7.41 ± 0.80	7.22 ± 0.76	7.12 ± 0.79	7.04 ± 0.82*
LAG, mm	0	2.43 ± 0.64	1.73 ± 0.70^※^	1.43 ± 0.70^※^	1.10 ± ±0.70^※^	0.92 ± 0.72^#^

MRD1, margin-to-reflex distance; PFH, palpebral fissure height; LAG, lagophthalmos

^*^,Compared with the preoperative levels, the value was significantly elevated at last Follow-up, *p *< 0.05

^※^,compared with immediate postoperative after surgery, the value of LAG at 1 months after surgery was statistically significant, *p *< 0.05

^※^,compared with 1 month after surgery, the value of LAG at 3 months after surgery was statistically significant, *p *< 0.05

^※^,compared with 3 month after surgery, the value of LAG at 6 months after surgery was statistically significant, *p *< 0.05

^#^,compared with 6 month after surgery, the value of LAG at last Follow-up

after surgery was not significant, *p *> 0.05

All patients had different degrees of lagophthalmos after surgery; as summarized in Table 3, the average values at the four time points were 2.43 ± 0.64 mm, 1.73 ± 0.70 mm, 1.43 ± 0.70 mm, 1.10 ± 0.70 mm, and 0.92 ± 0.72 mm, respectively. Over time, the degree of lagophthalmos gradually fell (*p*< 0.05) and tended to stabilize 6 months after surgery (*p*>0.05, Fig. 2c).

Postoperative complications included conjunctival prolapse and exposure keratitis. Conjunctival prolapse occurred in 3 eyes, 2 of which recovered after fixation with a deepening of the superior conjunctival vault, and one eye recovered after postoperative eye drops and bandages. Exposure keratitis occurred in 1 eye, which recovered 1 month after surgery following administration of eye drops, bandaging and soft contact lens use. No other complications occurred (Table 4).

**Table 4:** Postoperative effect, complication and satisfaction

Variable	Value
Postoperative effect	
Curative	30
Improvement	11
Ineffective	10
Overall effectiveness rate (%)	41/51(80.39%)
Complication	
Conjunctival prolapse	3
Exposure keratopathy	1
Total incidence rate (%)	4/51 (7.84%)
Satisfaction	
Highly satisfied	20
Generally satisfied	15
Dissatisfied	10
Overall satisfaction (%)	35/45 (77.78%)

**Fig. 3:**
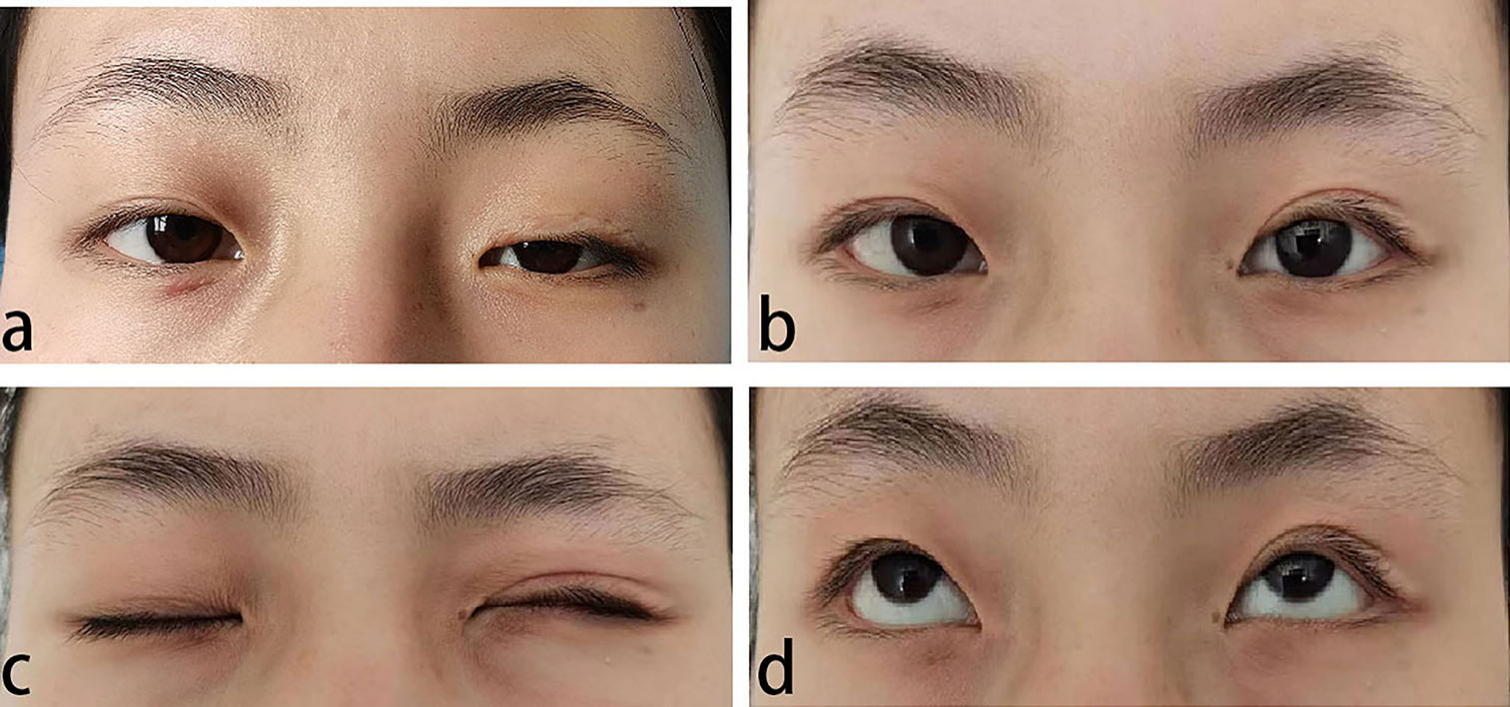
A 25-year-old girl was diagnosed with recurrent severe ptosis in left eye. Frontalis muscle flap suspension was performed on the left eye 10 years ago. Preoperative image of the second operation (**a**), with eyes open 1 years after CFS + LM suspension (**b**), and with image of eyes closed 1 years after surgery (**c**). The eye movement was normal (**d**)

**Fig. 4:**
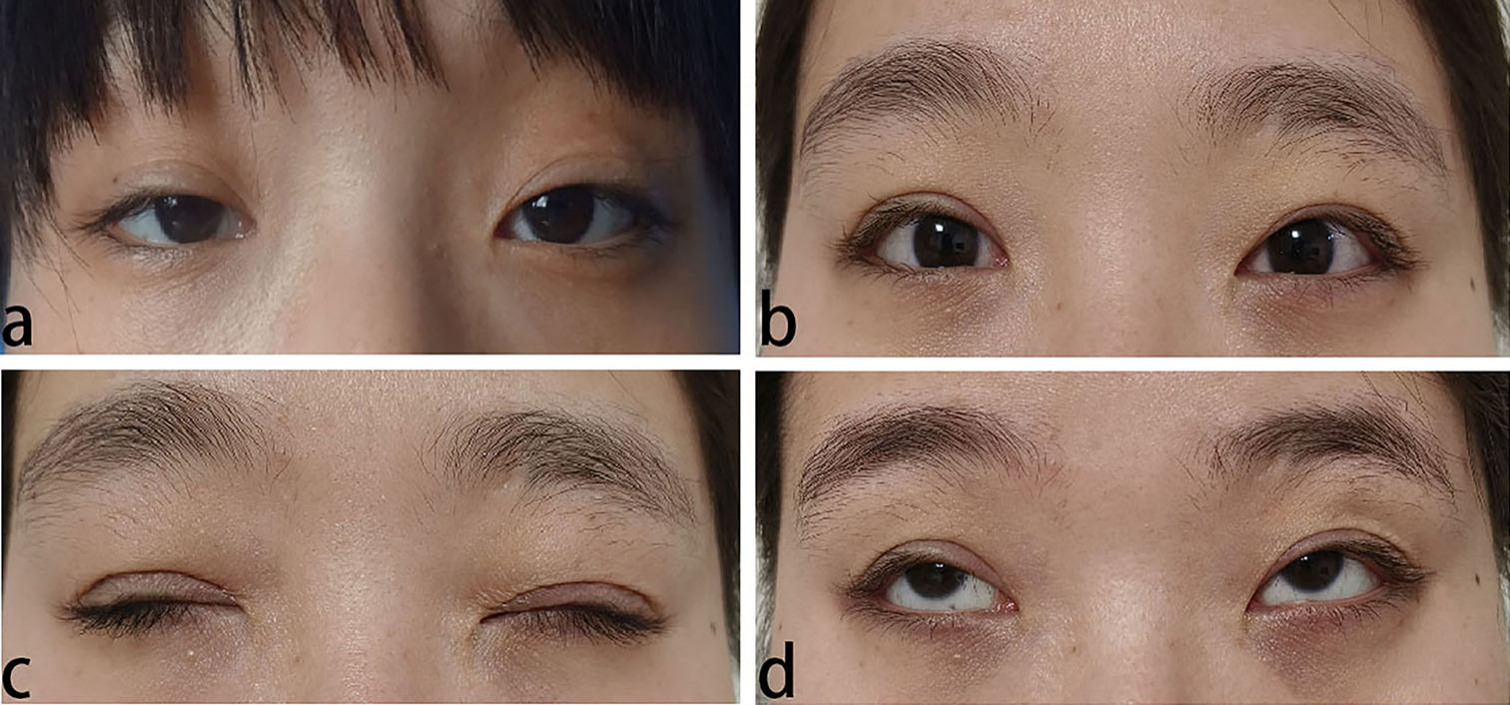
A 27-year-old girl was diagnosed with recurrent severe ptosis in right eye. Frontalis muscle flap suspension was performed on the right eye 20 years ago. Preoperative image of the second operation (**a**), with eyes open 1 years after CFS + LM suspension (**b**), and with image of eyes closed 1 years after surgery (**c**). The eye movement was normal (**d**)

Surgical outcomes were graded as curative in 30 patients, improved in 11 patients and ineffective in 10 patients at last follow-up. The overall improvement (curative+improved) rate was 80.39%. According to the patient satisfaction survey, 20 patients were satisfied with the outcomes, 15 patients were basically satisfied, and 10 patients were dissatisfied; the overall satisfaction rate was 77.78% (Table 4). Through both objective and subjective assessments, this modified surgery achieved satisfactory effects for patients with severe ptosis,good appearance was still obtained over one year without lagophthalmos after surgery, and eye movement was normal (Figs. 3 and 4).

## Discussion

Severe ptosis not only affects the appearance of patients but also often results in stimulus deprivation amblyopia due to the covering of the cornea by the upper eyelid, which can affect the development of visual function. Therefore, early surgical correction of severe ptosis is helpful for the treatment of amblyopia. However, the selection of surgical timing is very important for determining the postoperative effect. Owing to the immature development of the levator palpebral muscle and frontalis muscle, young patients are prone to ptosis recurrence after surgery, so these operations are typically reserved until the patients are over 2 years of age [13, 14]. For children less than 2 years of age, frontalis muscle suture suspension can be used, but long-term postoperative suture wear and the elasticity of the frontalis muscle will gradually worsen, eventually leading to recurrence. In this study, 9.80% of the patients had previously undergone frontalis suture suspension (Table 2). Some scholars believe that the optimal operative age for patients undergoing frontalis muscle flap suspension is over 5 years, as the frontalis muscle has fully developed by then [15, 16]. Over time, the elasticity and mobility of the frontalis muscle flap worsen, and the muscle gradually contributes less to lifting the upper eyelid. However, poor selection of surgical method also directly leads leading to postoperative recurrence. Levator-based procedures (levator advancement or resection) are usually performed for patients with mild and moderate ptosis, whereas frontalis suspension is used in patients with severe ptosis [17–20]. In this study, of the 45 patients (51 eyes) with severe ptosis, 41.18% had previously been treated with levator advancement, and 47.06% had been treated with frontalis suspension (Table 2).

According to classic literature, techniques where the levator function is below 5 mm rely on the frontalis muscle to take over the function, often utilizing autologous grafts like tensor fascia lata, whicn had achieved good clinical results [6, 21, 22]. However, for recurrent severe ptosis, if frontalis muscle flap suspension had previously been performed, the flap may be thin and fibrotic by the time of recurrence, and its effectiveness following repeat surgery will be poor. In addition, owing to the large difference between the suspension direction of the frontalis muscle and the physiological lifting direction of the upper eyelid, postoperative problems such as palpebral separation, long-term lagophthalmos, and an unnatural shape of the double eyelid may occur postoperatively [23]. Frontalis muscle suspension materials such as autologous fascia lata often increase damage to the donor area, whereas silicone strips and other synthetic materials not only are expensive but also may cause pain to the patient due to the long-term side effects of implantation of the material. For recurrent severe ptosis, if the levator muscle shortening technique had previously been performed, the remaining length of muscle may be insufficient to achieve the expected effect.

CFS suspension is a relatively new treatment method that has been favored by patients and clinicians in recent years [24]. However, in traditional CFS suspension, the CFS alone is used to suspend the tarsal plate. As the thickness of the CFS is only 1.1 ± 0.1 mm, the tissue is prone to tearing after surgery, potentially resulting in retraction of the upper eyelid. In severe cases, the ptosis may recur, leading to an overall poor postoperative effect. There is a lack of firm attachment points for CFS suspension alone, and the long term tension of the tissue cannot be maintained. Our previous research found that the elastin in the LM and CFS of severe ptosis in different age groups is abundant [25]. In the patients with poor muscle function, there was still a relatively rich amount of elastin, which provides theoretical support for the design and application of compound flaps. In this study, suture and fix the levator superior palpebral muscle with the CFS in the middle and upper parts of the tarsus to strengthen the elevated tarsus and reduce postoperative upper eyelid retraction. The difference between CFS suspension and frontalis muscle suspension is that the power source is different. The driving force of CFS comes predominantly from the superior rectus and, meantime, reserves the original function of the levator palpabrae muscle, which provides adequate force to lift upper eyelid [26]. With the help of the superior rectus to lift the upper eyelid and maintain certain movement, the height of the eyelid can be adjusted by the location of the CFS and tarsus. In addition, the contraction direction of the superior rectus is basically as same as LM. Compared with frontalis suspension, CFS suspension is more similar to physiological characteristics, avoiding the disadvantages of frontalis suspension [27, 28]. The emergence of CFS + LM suspension offers a new method of treatment for patients with severe ptosis. This study enrolled 45 patients (51 eyes) with recurrent severe ptosis. These patients underwent CFS + LM suspension, achieving a total postoperative effective rate of 80.39%, and a total degree of patient satisfaction of 74.47%, the latter of which is slightly lower than the 81.13% and 93.3%, respectively, from previous studies in which patients were treated with CFS + LM suspension for the first time [10, 29]. It was considered to be associated with local tissue scarring from the initial operation. Common complications, such as conjunctival prolapse, entropion, eyelid deformity, and exposure keratitis, have been observed. In this study, only 4 eyes experienced complications, for a rate of 7.84%, which is consistent with the rates in previous publications (9.43%) [29]. The average lagophthalmos was 0.92 ± 0.72 mm at the last follow-up.

In Asians, The normal height of the palpebral fissure is 8.2 mm in women and 8.5 mm in men. In this study, although the position of the upper eyelid and the height of the palpebral fissure gradually decreased over time after surgery, the latter was 7.04 ± 0.82 mm at the last follow-up, which, although lower than the average normal height of palpebral fissure in Asians, was better than the preoperative value (*p*< 0.05).

This research, however, is subject to several limitations. Firstly, the sample size of this study is small and the observation time is short, which may be biased in the judgment of efficacy. It is necessary to increase the sample size and extend the observation time in future studies to carry out a more comprehensive and in-depth study; Secondly, no control group was set up in this study. CFS + LM suspensionis a new surgical method for the treatment of severe ptosis, traditional frontal muscle flap suspension group should be set up for the treatment of recurrent severe ptosis, and the clinical efficacy, complications, satisfaction and other aspects of the two operations can be easily and fully compared, so that the research can have a more accurate clinical conclusion.

In summary, for recurrent severe ptosis, CFS + LM suspension described in this study not only is in line with the anatomy and physiological structure of the upper lid when the eye is open but also is easy to perform, causes minimal trauma, and has good treatment effects and few complications. This procedure should be widely adopted in clinical practice.

## Supplementary Information

Below is the link to the electronic supplementary material.

Supplementary file1 (MP4 42166 KB)

## Data Availability

The data that support the findings of this study are available from the corresponding author.
